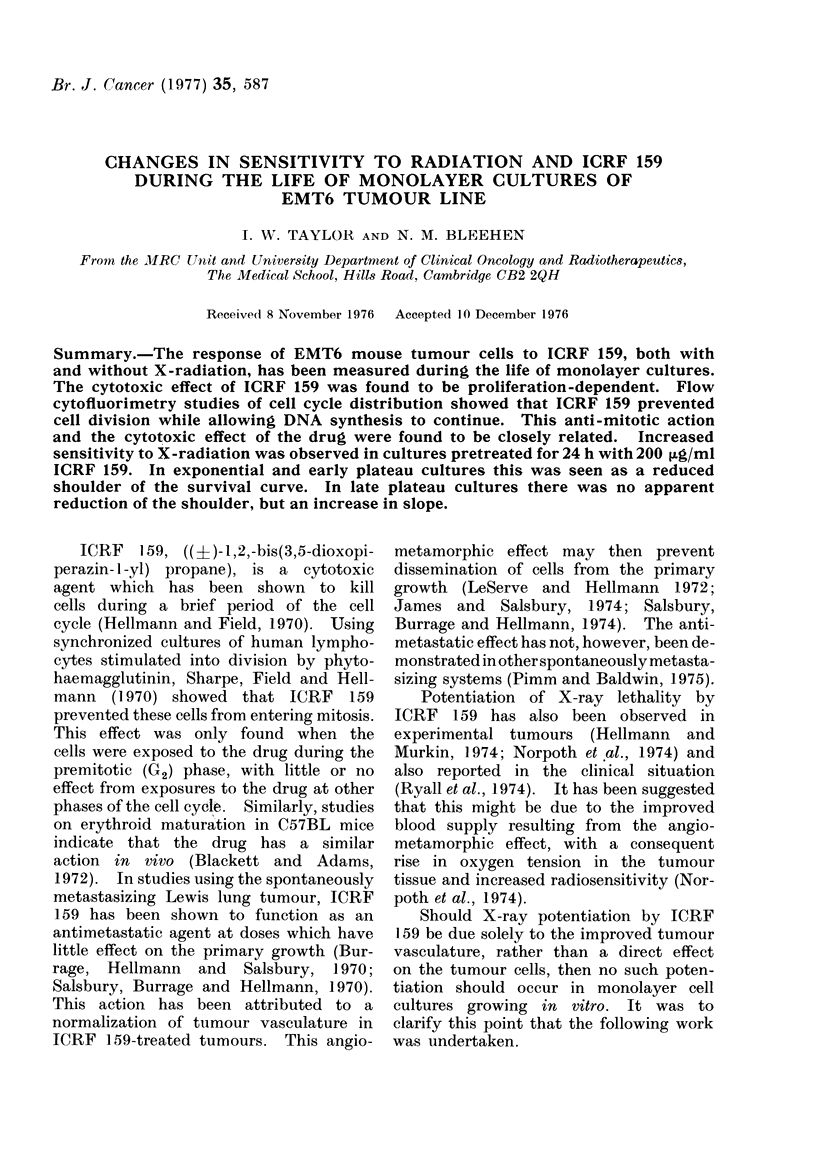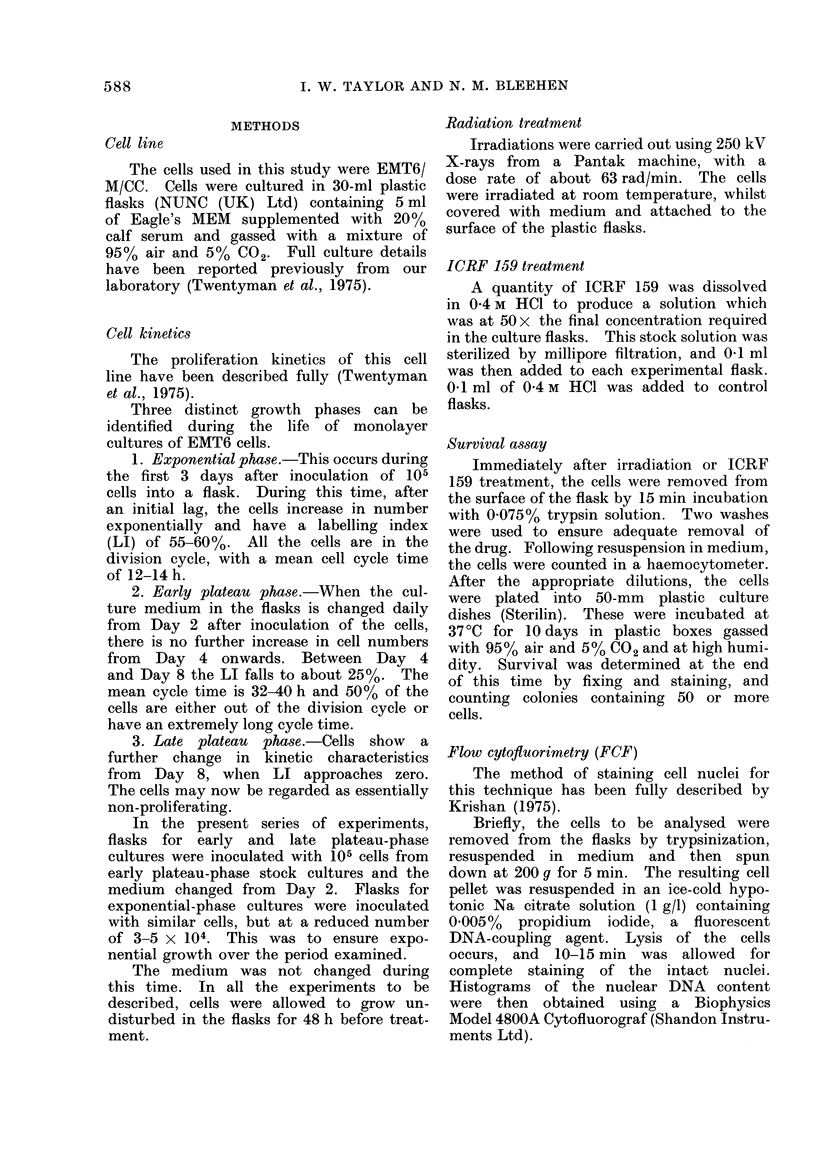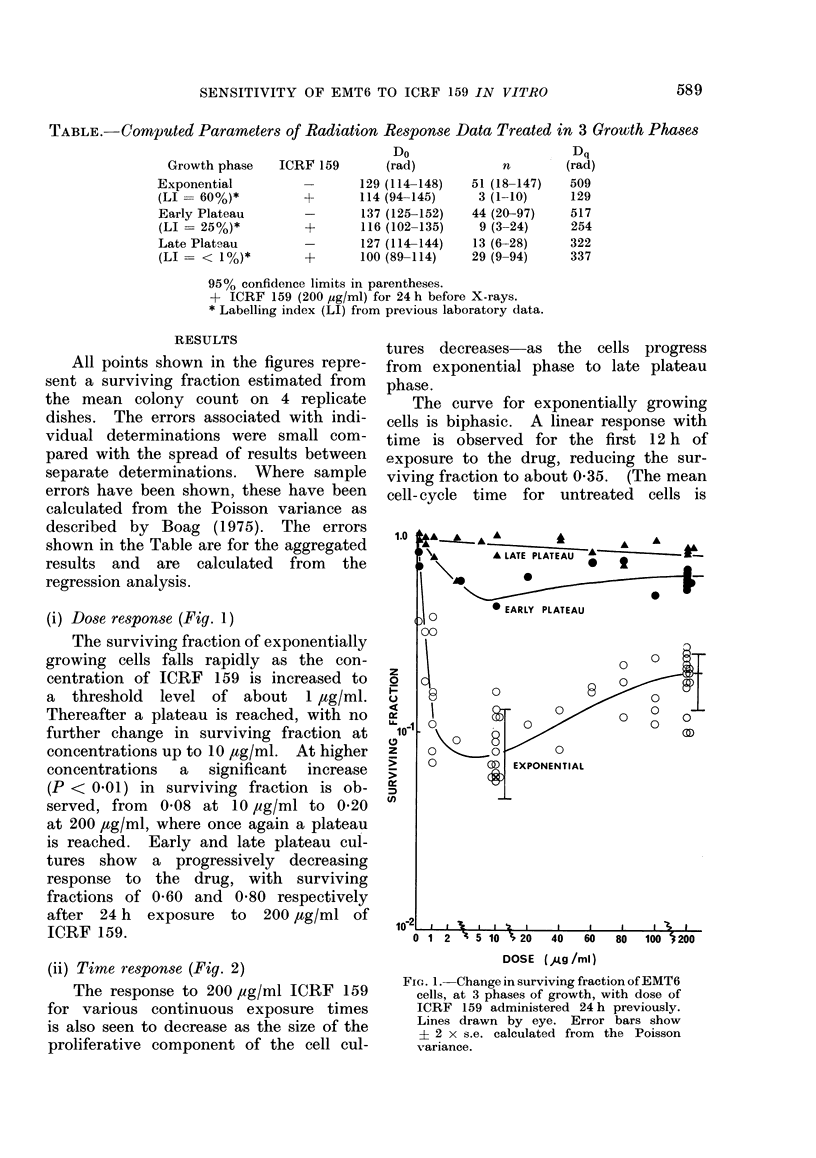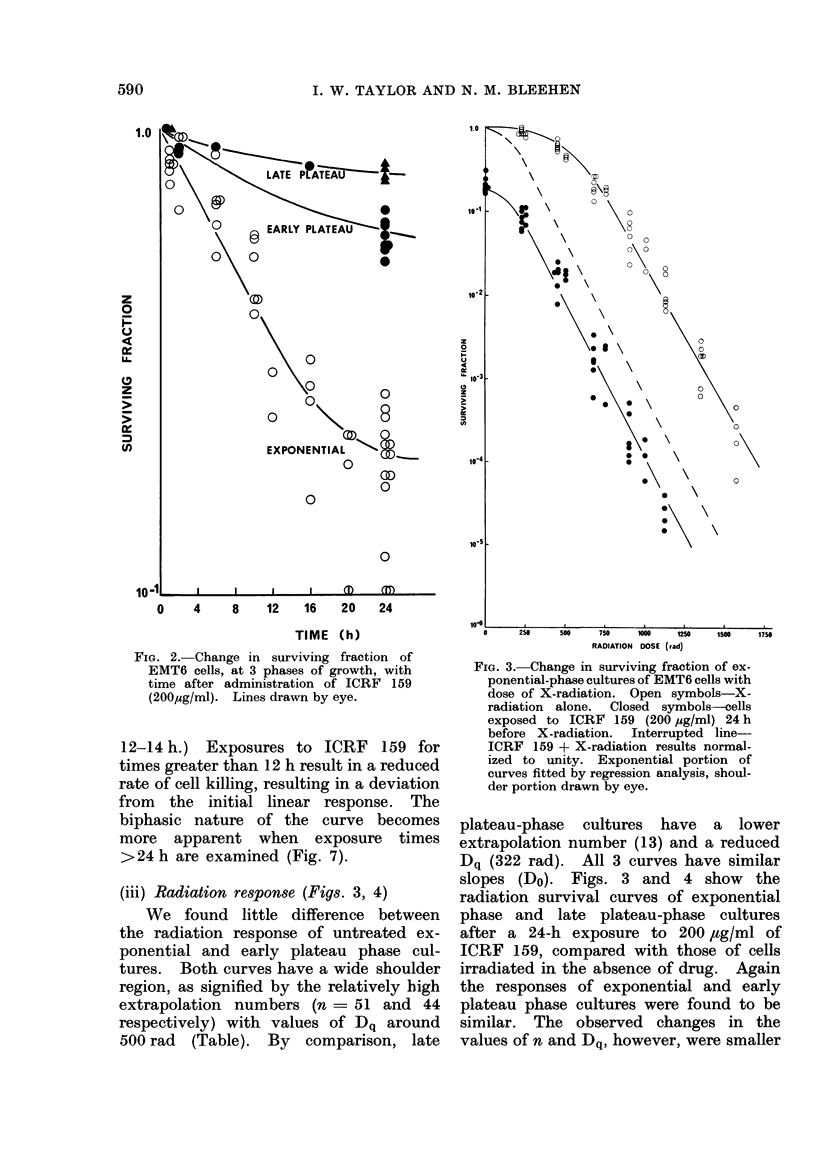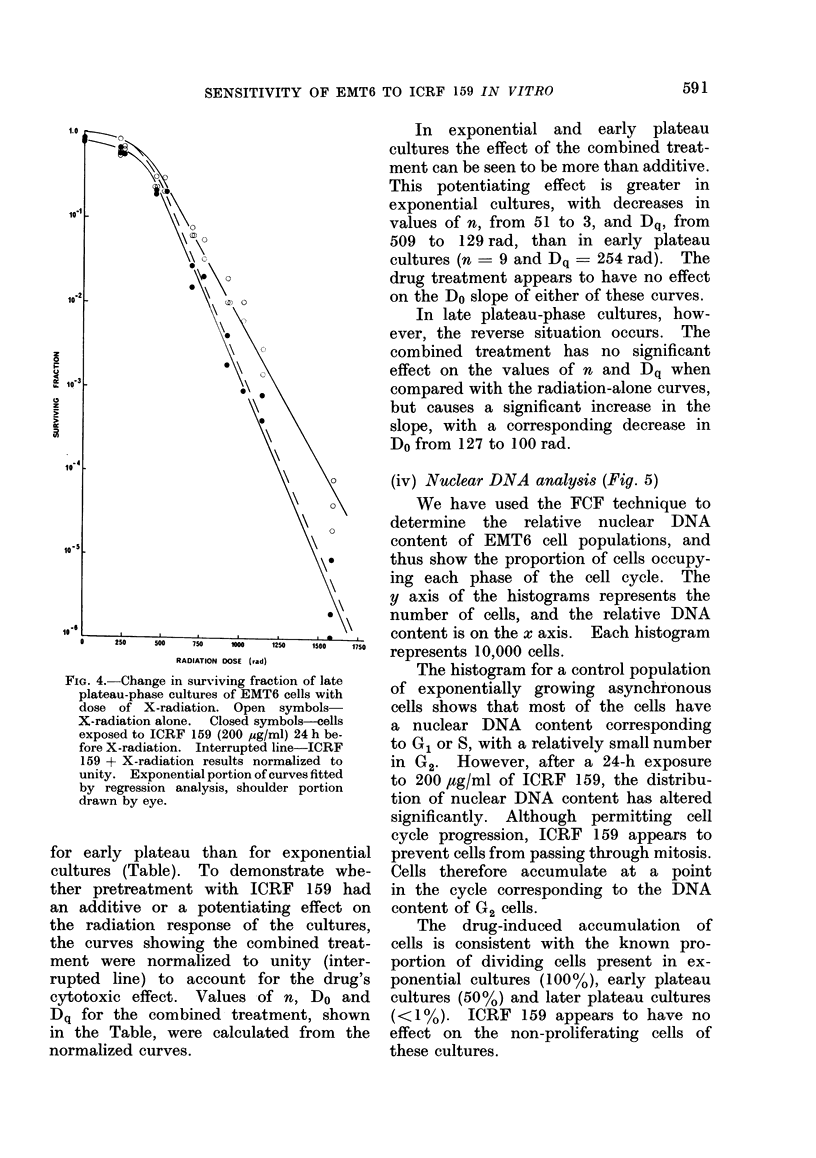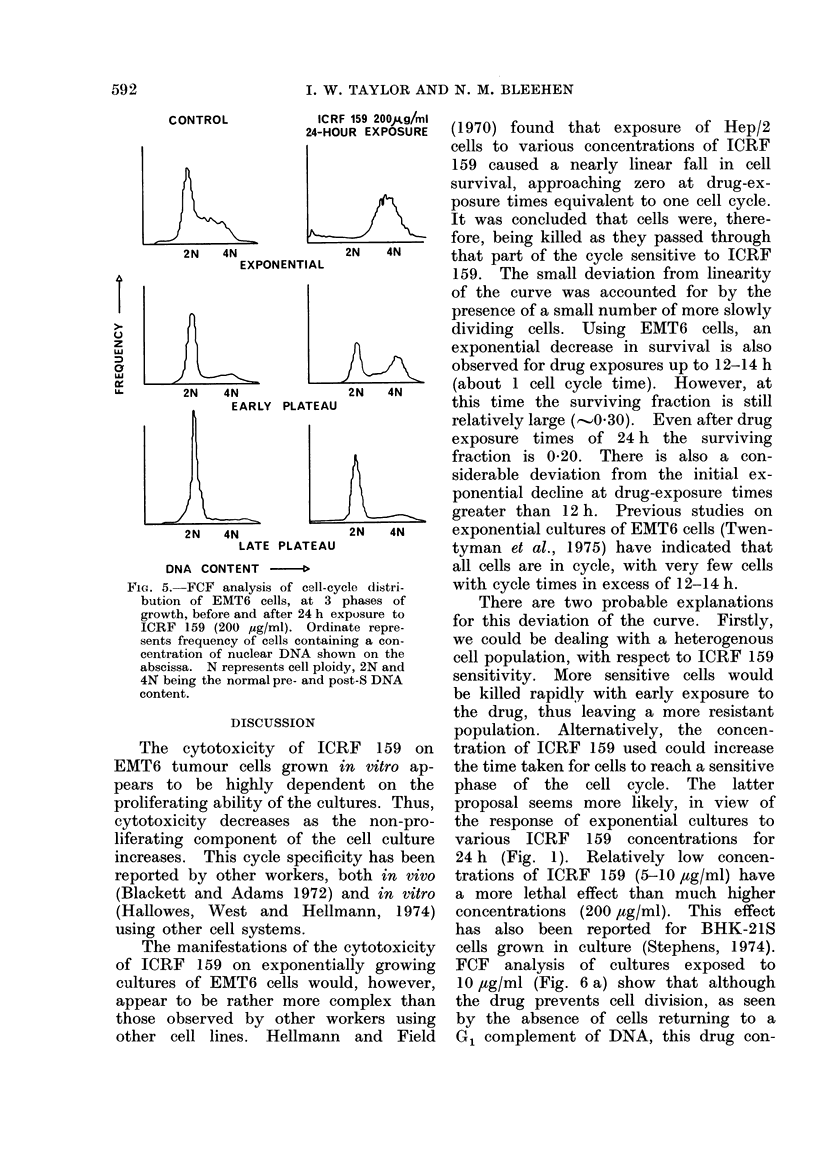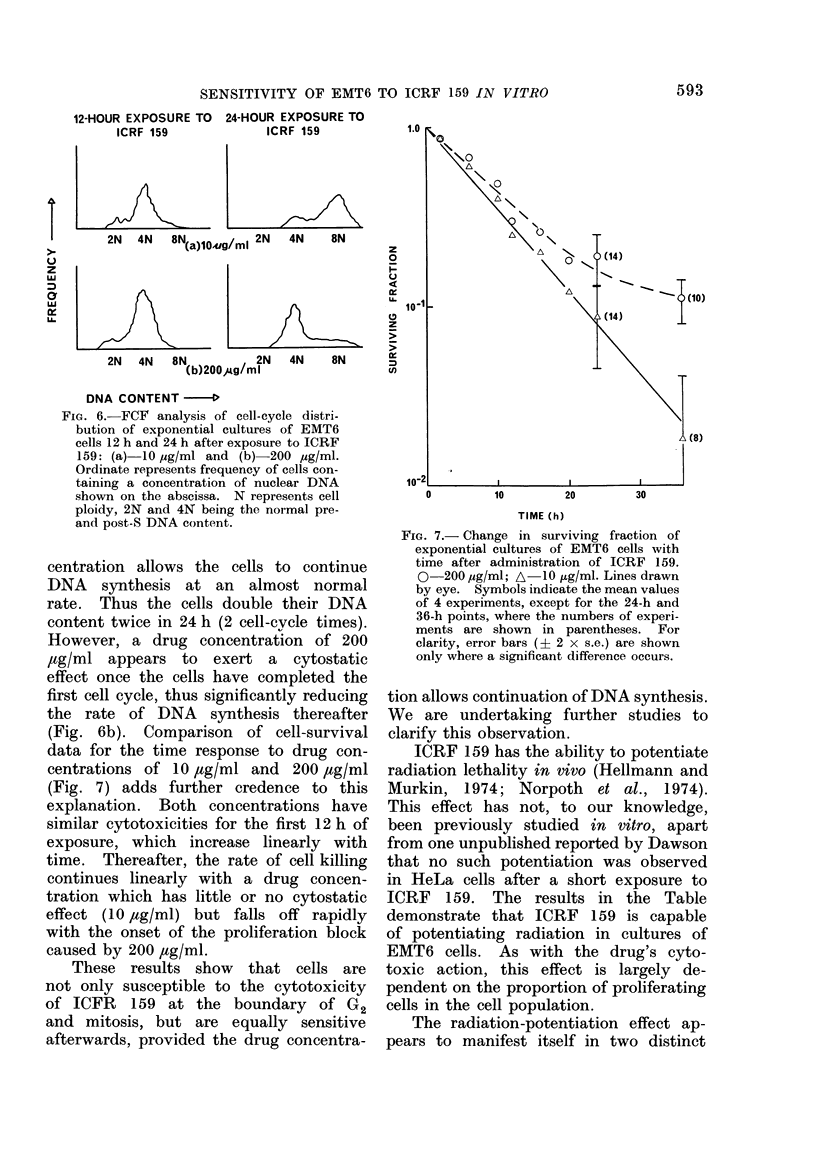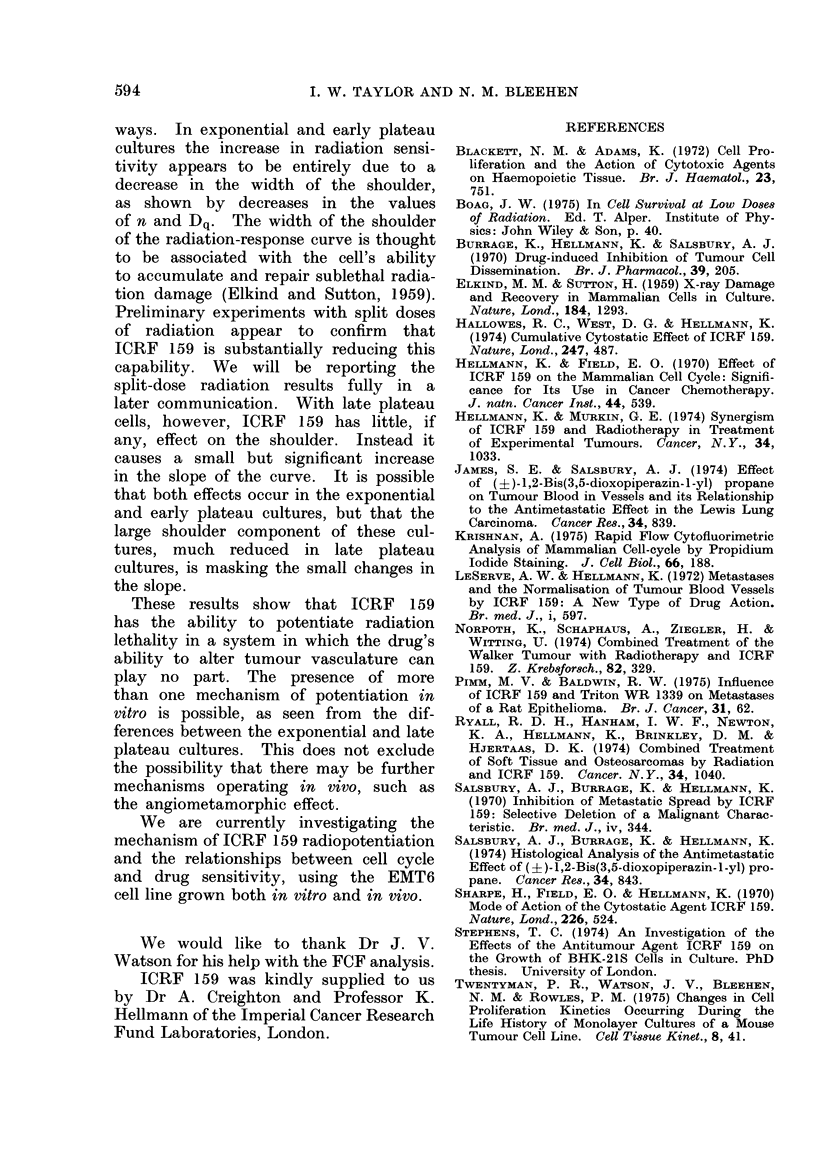# Changes in sensitivity to radiation and ICRF 159 during the life of monolayer cultures of EMT6 tumour line.

**DOI:** 10.1038/bjc.1977.92

**Published:** 1977-05

**Authors:** I. W. Taylor, N. M. Bleehen

## Abstract

The response of EMT6 mouse tumour cells to ICRF 159, both with and without X-radiation, has been measured during the life of monolayer cultures. The cytotoxic effect of ICRF 159 was found to be proliferation-dependent. Flow cytofluorimetry studies of cell cycle distribution showed that ICRF 159 prevented cell division while allowing DNA synthesis to continue. This anti-mitotic action and the cytotoxic effect of the drug were found to be closely related. Increased sensitivity to X-radiation was observed in cultures pretreated for 24 h with 200 microgram/ml ICRF 159 In exponential and early plateau cultures this was seen as a reduced shoulder of the survival curve. In late plateau cultures there was no apparent reduction of the shoulder, but an increase in slope.


*Br. J. Cancer* (1977) **35**, 587

### CHANGES IN SENSITIVITY TO RADIATION AND ICRF 159 DURING THE LIFE OF MONOLAYER CULTURES OF EMT6 TUMOUR LINE

I. W. TAYLOR AND N. M. BLEEHEN

*From the MRC Unit and University Department of Clinical Oncology and Radiotherapeutics, The Medical School, Hills Road, Cambridge CB2 2QH*

Received 8 November 1976 Accepted 10 December 1976

**Summary.—The response of EMT6 mouse tumour cells to ICRF 159, both with and without X-radiation, has been measured during the life of monolayer cultures. The cytotoxic effect of ICRF 159 was found to be proliferation-dependent. Flow cytofluorimetry studies of cell cycle distribution showed that ICRF 159 prevented cell division while allowing DNA synthesis to continue. This anti-mitotic action and the cytotoxic effect of the drug were found to be closely related. Increased sensitivity to X-radiation was observed in cultures pretreated for 24 h with 200 μg/ml ICRF 159. In exponential and early plateau cultures this was seen as a reduced shoulder of the survival curve. In late plateau cultures there was no apparent reduction of the shoulder, but an increase in slope.**

ICRF 159, ((±)-1,2,-bis(3,5-dioxopiperazin-1-yl) propane), is a cytotoxic agent which has been shown to kill cells during a brief period of the cell cycle (Hellmann and Field, 1970). Using synchronized cultures of human lymphocytes stimulated into division by phytohaemagglutinin, Sharpe, Field and Hellmann (1970) showed that ICRF 159 prevented these cells from entering mitosis. This effect was only found when the cells were exposed to the drug during the premitotic ($G_2$) phase, with little or no effect from exposures to the drug at other phases of the cell cycle. Similarly, studies on erythroid maturation in C57BL mice indicate that the drug has a similar action *in vivo* (Blackett and Adams, 1972). In studies using the spontaneously metastasizing Lewis lung tumour, ICRF 159 has been shown to function as an antimetastatic agent at doses which have little effect on the primary growth (Burrage, Hellmann and Salsbury, 1970; Salsbury, Burrage and Hellmann, 1970). This action has been attributed to a normalization of tumour vasculature in ICRF 159-treated tumours. This angiometamorphic effect may then prevent dissemination of cells from the primary growth (LeServe and Hellmann 1972; James and Salsbury, 1974; Salsbury, Burrage and Hellmann, 1974). The antimetastatic effect has not, however, been demonstrated in other spontaneously metastasizing systems (Pimm and Baldwin, 1975).

Potentiation of X-ray lethality by ICRF 159 has also been observed in experimental tumours (Hellmann and Murkin, 1974; Norpoth *et al.*, 1974) and also reported in the clinical situation (Ryall *et al.*, 1974). It has been suggested that this might be due to the improved blood supply resulting from the angiometamorphic effect, with a consequent rise in oxygen tension in the tumour tissue and increased radiosensitivity (Norpoth *et al.*, 1974).

Should X-ray potentiation by ICRF 159 be due solely to the improved tumour vasculature, rather than a direct effect on the tumour cells, then no such potentiation should occur in monolayer cell cultures growing *in vitro*. It was to clarify this point that the following work was undertaken.

### METHODS

### *Cell line*

The cells used in this study were EMT6/M/CC. Cells were cultured in 30-ml plastic flasks (NUNC (UK) Ltd) containing 5 ml of Eagle's MEM supplemented with 20% calf serum and gassed with a mixture of 95% air and 5% $CO_2$. Full culture details have been reported previously from our laboratory (Twentyman *et al.*, 1975).

### *Cell kinetics*

The proliferation kinetics of this cell line have been described fully (Twentyman *et al.*, 1975).

Three distinct growth phases can be identified during the life of monolayer cultures of EMT6 cells.

1. *Exponential phase.*—This occurs during the first 3 days after inoculation of $10^5$ cells into a flask. During this time, after an initial lag, the cells increase in number exponentially and have a labelling index (LI) of 55–60%. All the cells are in the division cycle, with a mean cell cycle time of 12–14 h.

2. *Early plateau phase.*—When the culture medium in the flasks is changed daily from Day 2 after inoculation of the cells, there is no further increase in cell numbers from Day 4 onwards. Between Day 4 and Day 8 the LI falls to about 25%. The mean cycle time is 32–40 h and 50% of the cells are either out of the division cycle or have an extremely long cycle time.

3. *Late plateau phase.*—Cells show a further change in kinetic characteristics from Day 8, when LI approaches zero. The cells may now be regarded as essentially non-proliferating.

In the present series of experiments, flasks for early and late plateau-phase cultures were inoculated with $10^5$ cells from early plateau-phase stock cultures and the medium changed from Day 2. Flasks for exponential-phase cultures were inoculated with similar cells, but at a reduced number of $3–5 \times 10^4$. This was to ensure exponential growth over the period examined.

The medium was not changed during this time. In all the experiments to be described, cells were allowed to grow undisturbed in the flasks for 48 h before treatment.

### *Radiation treatment*

Irradiations were carried out using 250 kV X-rays from a Pantak machine, with a dose rate of about 63 rad/min. The cells were irradiated at room temperature, whilst covered with medium and attached to the surface of the plastic flasks.

### *ICRF 159 treatment*

A quantity of ICRF 159 was dissolved in 0·4 M HCl to produce a solution which was at 50× the final concentration required in the culture flasks. This stock solution was sterilized by millipore filtration, and 0·1 ml was then added to each experimental flask. 0·1 ml of 0·4 M HCl was added to control flasks.

### *Survival assay*

Immediately after irradiation or ICRF 159 treatment, the cells were removed from the surface of the flask by 15 min incubation with 0·075% trypsin solution. Two washes were used to ensure adequate removal of the drug. Following resuspension in medium, the cells were counted in a haemocytometer. After the appropriate dilutions, the cells were plated into 50-mm plastic culture dishes (Sterilin). These were incubated at 37°C for 10 days in plastic boxes gassed with 95% air and 5% $CO_2$ and at high humidity. Survival was determined at the end of this time by fixing and staining, and counting colonies containing 50 or more cells.

### *Flow cytofluorimetry (FCF)*

The method of staining cell nuclei for this technique has been fully described by Krishan (1975).

Briefly, the cells to be analysed were removed from the flasks by trypsinization, resuspended in medium and then spun down at 200 *g* for 5 min. The resulting cell pellet was resuspended in an ice-cold hypotonic Na citrate solution (1 g/l) containing 0·005% propidium iodide, a fluorescent DNA-coupling agent. Lysis of the cells occurs, and 10–15 min was allowed for complete staining of the intact nuclei. Histograms of the nuclear DNA content were then obtained using a Biophysics Model 4800A Cytofluorograf (Shandon Instruments Ltd).

TABLE.—*Computed Parameters of Radiation Response Data Treated in 3 Growth Phases*

| Growth phase | ICRF 159 | $D_0$ (rad) | *n* | $D_q$ (rad) |
|---|---|---|---|---|
| Exponential | − | 129 (114–148) | 51 (18–147) | 509 |
| (LI = 60%)* | + | 114 (94–145) | 3 (1–10) | 129 |
| Early Plateau | − | 137 (125–152) | 44 (20–97) | 517 |
| (LI = 25%)* | + | 116 (102–135) | 9 (3–24) | 254 |
| Late Plateau | − | 127 (114–144) | 13 (6–28) | 322 |
| (LI = < 1%)* | + | 100 (89–114) | 29 (9–94) | 337 |

95% confidence limits in parentheses.
+ ICRF 159 (200 μg/ml) for 24 h before X-rays.
* Labelling index (LI) from previous laboratory data.

### RESULTS

All points shown in the figures represent a surviving fraction estimated from the mean colony count on 4 replicate dishes. The errors associated with individual determinations were small compared with the spread of results between separate determinations. Where sample errors have been shown, these have been calculated from the Poisson variance as described by Boag (1975). The errors shown in the Table are for the aggregated results and are calculated from the regression analysis.

### (i) *Dose response* (*Fig.* 1)

The surviving fraction of exponentially growing cells falls rapidly as the concentration of ICRF 159 is increased to a threshold level of about 1 μg/ml. Thereafter a plateau is reached, with no further change in surviving fraction at concentrations up to 10 μg/ml. At higher concentrations a significant increase ($P < 0{\cdot}01$) in surviving fraction is observed, from 0·08 at 10 μg/ml to 0·20 at 200 μg/ml, where once again a plateau is reached. Early and late plateau cultures show a progressively decreasing response to the drug, with surviving fractions of 0·60 and 0·80 respectively after 24 h exposure to 200 μg/ml of ICRF 159.

### (ii) *Time response* (*Fig.* 2)

The response to 200 μg/ml ICRF 159 for various continuous exposure times is also seen to decrease as the size of the proliferative component of the cell cultures decreases—as the cells progress from exponential phase to late plateau phase.

The curve for exponentially growing cells is biphasic. A linear response with time is observed for the first 12 h of exposure to the drug, reducing the surviving fraction to about 0·35. (The mean cell-cycle time for untreated cells is

FIG. 1.—Change in surviving fraction of EMT6 cells, at 3 phases of growth, with dose of ICRF 159 administered 24 h previously. Lines drawn by eye. Error bars show ± 2 × s.e. calculated from the Poisson variance.

Fig. 2.—Change in surviving fraction of EMT6 cells, at 3 phases of growth, with time after administration of ICRF 159 (200μg/ml). Lines drawn by eye.

Fig. 3.—Change in surviving fraction of exponential-phase cultures of EMT6 cells with dose of X-radiation. Open symbols—X-radiation alone. Closed symbols—cells exposed to ICRF 159 (200 μg/ml) 24 h before X-radiation. Interrupted line—ICRF 159 + X-radiation results normalized to unity. Exponential portion of curves fitted by regression analysis, shoulder portion drawn by eye.

12–14 h.) Exposures to ICRF 159 for times greater than 12 h result in a reduced rate of cell killing, resulting in a deviation from the initial linear response. The biphasic nature of the curve becomes more apparent when exposure times >24 h are examined (Fig. 7).

(iii) *Radiation response* (*Figs.* 3, 4)

We found little difference between the radiation response of untreated exponential and early plateau phase cultures. Both curves have a wide shoulder region, as signified by the relatively high extrapolation numbers ($n = 51$ and 44 respectively) with values of $D_q$ around 500 rad (Table). By comparison, late plateau-phase cultures have a lower extrapolation number (13) and a reduced $D_q$ (322 rad). All 3 curves have similar slopes ($D_0$). Figs. 3 and 4 show the radiation survival curves of exponential phase and late plateau-phase cultures after a 24-h exposure to 200 μg/ml of ICRF 159, compared with those of cells irradiated in the absence of drug. Again the responses of exponential and early plateau phase cultures were found to be similar. The observed changes in the values of $n$ and $D_q$, however, were smaller

FIG. 4.—Change in surviving fraction of late plateau-phase cultures of EMT6 cells with dose of X-radiation. Open symbols—X-radiation alone. Closed symbols—cells exposed to ICRF 159 (200 $\mu$g/ml) 24 h before X-radiation. Interrupted line—ICRF 159 + X-radiation results normalized to unity. Exponential portion of curves fitted by regression analysis, shoulder portion drawn by eye.

for early plateau than for exponential cultures (Table). To demonstrate whether pretreatment with ICRF 159 had an additive or a potentiating effect on the radiation response of the cultures, the curves showing the combined treatment were normalized to unity (interrupted line) to account for the drug's cytotoxic effect. Values of $n$, $D_0$ and $D_q$ for the combined treatment, shown in the Table, were calculated from the normalized curves.

In exponential and early plateau cultures the effect of the combined treatment can be seen to be more than additive. This potentiating effect is greater in exponential cultures, with decreases in values of $n$, from 51 to 3, and $D_q$, from 509 to 129 rad, than in early plateau cultures ($n = 9$ and $D_q = 254$ rad). The drug treatment appears to have no effect on the $D_0$ slope of either of these curves.

In late plateau-phase cultures, however, the reverse situation occurs. The combined treatment has no significant effect on the values of $n$ and $D_q$ when compared with the radiation-alone curves, but causes a significant increase in the slope, with a corresponding decrease in $D_0$ from 127 to 100 rad.

(iv) *Nuclear DNA analysis* (*Fig.* 5)

We have used the FCF technique to determine the relative nuclear DNA content of EMT6 cell populations, and thus show the proportion of cells occupying each phase of the cell cycle. The $y$ axis of the histograms represents the number of cells, and the relative DNA content is on the $x$ axis. Each histogram represents 10,000 cells.

The histogram for a control population of exponentially growing asynchronous cells shows that most of the cells have a nuclear DNA content corresponding to $G_1$ or S, with a relatively small number in $G_2$. However, after a 24-h exposure to 200 $\mu$g/ml of ICRF 159, the distribution of nuclear DNA content has altered significantly. Although permitting cell cycle progression, ICRF 159 appears to prevent cells from passing through mitosis. Cells therefore accumulate at a point in the cycle corresponding to the DNA content of $G_2$ cells.

The drug-induced accumulation of cells is consistent with the known proportion of dividing cells present in exponential cultures (100%), early plateau cultures (50%) and later plateau cultures (<1%). ICRF 159 appears to have no effect on the non-proliferating cells of these cultures.

FIG. 5.—FCF analysis of cell-cycle distribution of EMT6 cells, at 3 phases of growth, before and after 24 h exposure to ICRF 159 (200 μg/ml). Ordinate represents frequency of cells containing a concentration of nuclear DNA shown on the abscissa. N represents cell ploidy, 2N and 4N being the normal pre- and post-S DNA content.

### DISCUSSION

The cytotoxicity of ICRF 159 on EMT6 tumour cells grown *in vitro* appears to be highly dependent on the proliferating ability of the cultures. Thus, cytotoxicity decreases as the non-proliferating component of the cell culture increases. This cycle specificity has been reported by other workers, both *in vivo* (Blackett and Adams 1972) and *in vitro* (Hallowes, West and Hellmann, 1974) using other cell systems.

The manifestations of the cytotoxicity of ICRF 159 on exponentially growing cultures of EMT6 cells would, however, appear to be rather more complex than those observed by other workers using other cell lines. Hellmann and Field (1970) found that exposure of Hep/2 cells to various concentrations of ICRF 159 caused a nearly linear fall in cell survival, approaching zero at drug-exposure times equivalent to one cell cycle. It was concluded that cells were, therefore, being killed as they passed through that part of the cycle sensitive to ICRF 159. The small deviation from linearity of the curve was accounted for by the presence of a small number of more slowly dividing cells. Using EMT6 cells, an exponential decrease in survival is also observed for drug exposures up to 12–14 h (about 1 cell cycle time). However, at this time the surviving fraction is still relatively large (~0·30). Even after drug exposure times of 24 h the surviving fraction is 0·20. There is also a considerable deviation from the initial exponential decline at drug-exposure times greater than 12 h. Previous studies on exponential cultures of EMT6 cells (Twentyman *et al.*, 1975) have indicated that all cells are in cycle, with very few cells with cycle times in excess of 12–14 h.

There are two probable explanations for this deviation of the curve. Firstly, we could be dealing with a heterogenous cell population, with respect to ICRF 159 sensitivity. More sensitive cells would be killed rapidly with early exposure to the drug, thus leaving a more resistant population. Alternatively, the concentration of ICRF 159 used could increase the time taken for cells to reach a sensitive phase of the cell cycle. The latter proposal seems more likely, in view of the response of exponential cultures to various ICRF 159 concentrations for 24 h (Fig. 1). Relatively low concentrations of ICRF 159 (5–10 μg/ml) have a more lethal effect than much higher concentrations (200 μg/ml). This effect has also been reported for BHK-21S cells grown in culture (Stephens, 1974). FCF analysis of cultures exposed to 10 μg/ml (Fig. 6 a) show that although the drug prevents cell division, as seen by the absence of cells returning to a $G_1$ complement of DNA, this drug con-

FIG. 6.—FCF analysis of cell-cycle distribution of exponential cultures of EMT6 cells 12 h and 24 h after exposure to ICRF 159: (a)—10 μg/ml and (b)—200 μg/ml. Ordinate represents frequency of cells containing a concentration of nuclear DNA shown on the abscissa. N represents cell ploidy, 2N and 4N being the normal pre- and post-S DNA content.

FIG. 7.— Change in surviving fraction of exponential cultures of EMT6 cells with time after administration of ICRF 159. ○—200 μg/ml; △—10 μg/ml. Lines drawn by eye. Symbols indicate the mean values of 4 experiments, except for the 24-h and 36-h points, where the numbers of experiments are shown in parentheses. For clarity, error bars (± 2 × s.e.) are shown only where a significant difference occurs.

centration allows the cells to continue DNA synthesis at an almost normal rate. Thus the cells double their DNA content twice in 24 h (2 cell-cycle times). However, a drug concentration of 200 μg/ml appears to exert a cytostatic effect once the cells have completed the first cell cycle, thus significantly reducing the rate of DNA synthesis thereafter (Fig. 6b). Comparison of cell-survival data for the time response to drug concentrations of 10 μg/ml and 200 μg/ml (Fig. 7) adds further credence to this explanation. Both concentrations have similar cytotoxicities for the first 12 h of exposure, which increase linearly with time. Thereafter, the rate of cell killing continues linearly with a drug concentration which has little or no cytostatic effect (10 μg/ml) but falls off rapidly with the onset of the proliferation block caused by 200 μg/ml.

These results show that cells are not only susceptible to the cytotoxicity of ICFR 159 at the boundary of $G_2$ and mitosis, but are equally sensitive afterwards, provided the drug concentration allows continuation of DNA synthesis. We are undertaking further studies to clarify this observation.

ICRF 159 has the ability to potentiate radiation lethality *in vivo* (Hellmann and Murkin, 1974; Norpoth *et al.*, 1974). This effect has not, to our knowledge, been previously studied *in vitro*, apart from one unpublished reported by Dawson that no such potentiation was observed in HeLa cells after a short exposure to ICRF 159. The results in the Table demonstrate that ICRF 159 is capable of potentiating radiation in cultures of EMT6 cells. As with the drug's cytotoxic action, this effect is largely dependent on the proportion of proliferating cells in the cell population.

The radiation-potentiation effect appears to manifest itself in two distinct ways. In exponential and early plateau cultures the increase in radiation sensitivity appears to be entirely due to a decrease in the width of the shoulder, as shown by decreases in the values of $n$ and $D_q$. The width of the shoulder of the radiation-response curve is thought to be associated with the cell's ability to accumulate and repair sublethal radiation damage (Elkind and Sutton, 1959). Preliminary experiments with split doses of radiation appear to confirm that ICRF 159 is substantially reducing this capability. We will be reporting the split-dose radiation results fully in a later communication. With late plateau cells, however, ICRF 159 has little, if any, effect on the shoulder. Instead it causes a small but significant increase in the slope of the curve. It is possible that both effects occur in the exponential and early plateau cultures, but that the large shoulder component of these cultures, much reduced in late plateau cultures, is masking the small changes in the slope.

These results show that ICRF 159 has the ability to potentiate radiation lethality in a system in which the drug's ability to alter tumour vasculature can play no part. The presence of more than one mechanism of potentiation *in vitro* is possible, as seen from the differences between the exponential and late plateau cultures. This does not exclude the possibility that there may be further mechanisms operating *in vivo*, such as the angiometamorphic effect.

We are currently investigating the mechanism of ICRF 159 radiopotentiation and the relationships between cell cycle and drug sensitivity, using the EMT6 cell line grown both *in vitro* and *in vivo*.

We would like to thank Dr J. V. Watson for his help with the FCF analysis.

ICRF 159 was kindly supplied to us by Dr A. Creighton and Professor K. Hellmann of the Imperial Cancer Research Fund Laboratories, London.

REFERENCES

Blackett, N. M. & Adams, K. (1972) Cell Proliferation and the Action of Cytotoxic Agents on Haemopoietic Tissue. *Br. J. Haematol.*, **23**, 751.

Boag, J. W. (1975) In *Cell Survival at Low Doses of Radiation*. Ed. T. Alper. Institute of Physics: John Wiley & Son, p. 40.

Burrage, K., Hellmann, K. & Salsbury, A. J. (1970) Drug-induced Inhibition of Tumour Cell Dissemination. *Br. J. Pharmacol.*, **39**, 205.

Elkind, M. M. & Sutton, H. (1959) X-ray Damage and Recovery in Mammalian Cells in Culture. *Nature, Lond.*, **184**, 1293.

Hallowes, R. C., West, D. G. & Hellmann, K. (1974) Cumulative Cytostatic Effect of ICRF 159. *Nature, Lond.*, **247**, 487.

Hellmann, K. & Field, E. O. (1970) Effect of ICRF 159 on the Mammalian Cell Cycle: Significance for Its Use in Cancer Chemotherapy. *J. natn. Cancer Inst.*, **44**, 539.

Hellmann, K. & Murkin, G. E. (1974) Synergism of ICRF 159 and Radiotherapy in Treatment of Experimental Tumours. *Cancer, N.Y.*, **34**, 1033.

James, S. E. & Salsbury, A. J. (1974) Effect of (±)-1,2-Bis(3,5-dioxopiperazin-1-yl) propane on Tumour Blood in Vessels and its Relationship to the Antimetastatic Effect in the Lewis Lung Carcinoma. *Cancer Res.*, **34**, 839.

Krishnan, A. (1975) Rapid Flow Cytofluorimetric Analysis of Mammalian Cell-cycle by Propidium Iodide Staining. *J. Cell Biol.*, **66**, 188.

LeServe, A. W. & Hellmann, K. (1972) Metastases and the Normalisation of Tumour Blood Vessels by ICRF 159: A New Type of Drug Action. *Br. med. J.*, i, 597.

Norpoth, K., Schaphaus, A., Ziegler, H. & Witting, U. (1974) Combined Treatment of the Walker Tumour with Radiotherapy and ICRF 159. *Z. Krebsforsch.*, **82**, 329.

Pimm, M. V. & Baldwin, R. W. (1975) Influence of ICRF 159 and Triton WR 1339 on Metastases of a Rat Epithelioma. *Br. J. Cancer*, **31**, 62.

Ryall, R. D. H., Hanham, I. W. F., Newton, K. A., Hellmann, K., Brinkley, D. M. & Hjertaas, D. K. (1974) Combined Treatment of Soft Tissue and Osteosarcomas by Radiation and ICRF 159. *Cancer. N.Y.*, **34**, 1040.

Salsbury, A. J., Burrage, K. & Hellmann, K. (1970) Inhibition of Metastatic Spread by ICRF 159: Selective Deletion of a Malignant Characteristic. *Br. med. J.*, iv, 344.

Salsbury, A. J., Burrage, K. & Hellmann, K. (1974) Histological Analysis of the Antimetastatic Effect of (±)-1,2-Bis(3,5-dioxopiperazin-1-yl) propane. *Cancer Res.*, **34**, 843.

Sharpe, H., Field, E. O. & Hellmann, K. (1970) Mode of Action of the Cytostatic Agent ICRF 159. *Nature, Lond.*, **226**, 524.

Stephens, T. C. (1974) An Investigation of the Effects of the Antitumour Agent ICRF 159 on the Growth of BHK-21S Cells in Culture. PhD thesis. University of London.

Twentyman, P. R., Watson, J. V., Bleehen, N. M. & Rowles, P. M. (1975) Changes in Cell Proliferation Kinetics Occurring During the Life History of Monolayer Cultures of a Mouse Tumour Cell Line. *Cell Tissue Kinet.*, **8**, 41.